# Remarks on the Influence of Acids in Promoting Salivation, Communicated in a Letter to Dr. Drake

**Published:** 1830

**Authors:** Thomas Townsend

**Affiliations:** Massilon, Stark Co. Ohio


Art.—V. *Remarks on the influence of Acids in promoting Salivation.* Communicated in a letter to Dr. Drake.

Dear Sir.—I observed in the April No. of the "Western Journal of Medical and Physical Sciences," an article from the North American Med. and Surg. Journal, noticing a letter of Dr. Harty, of Dublin, in which it is stated that the use of the sulphate of quinine accelerates mercurial salivation; and frequently induces calomel to excite ptyalism, when it probably would not otherwise have occurred.

In reflecting on this subject, I am inclined to think that Dr. Harty has not made the requisite observation on the use of the article. I have been in the practice of using the sulphate of quinine pretty extensively for several years, during the administration of calomel, and have observed no such effect as that of accelerating mercurial salivation. Besides, Dr. H. has not stated *his manner of preparing and exhibiting the quinine*, which is certainly very important in forming an opinion on the subject. If Dr. H. dissolved the quinine in water, by adding a small quantity of diluted sulphuric acid, as is a very common practice, ptyalism, when calomel or any other preparation of mercury had been used, would be a very probable consequence, as I have often witnessed; but the salivation in this case, would be owing to the acid in the mixture, and not to the quinine.

I have observed for a number of years, that acids of any kind will cause calomel to affect the mouth, when no such effect would have resulted from its use, had the acids been entirely avoided.

Bilious remittent fevers prevailed extensively, during the summer of 1828, in this place and the surrounding country, and my brother and myself were in the daily use of calomel, as a cathartic, and quinine as a tonic, in the later stages of the disease; yet we had not more than five or six cases of sore mouth, and these were brought about by the use of acids—lemonade, vinegar, or soda powders, in which there was an excess of acid, or pickles, &c. We are in the practice of giving calomel liberally, without having any thing like ptyalism in any case where we do not wish to produce it, and can get our patients rigidly to follow directions.

I am of opinion, that in remittent fevers, mercury, when its action is determined to the glands about the mouth, becomes, instead of a salutary remedy, *a local irritant*. If this opinion be true, a salivation ought not to be produced by mercury, in this disease, unless we feel confident that we can produce, at the same time, *a mercurial fever*, which shall supercede and take the place of the original disease. Even in this case it is questionable, whether the indication may not be better answered by a different course. I have long since adopted the opinion, that *we may, with proper care, have all the advantages which mercury can afford, without its inconveniences*, in every description of fever calling for its use. With these views, I have prescribed it almost habitually, both as a cathartic and alterative, only using the precaution to direct my patients to avoid every kind of acid. The prejudices, and almost insuperable objections which many people have to the use of calomel, seem to have had their origin in the inconveniences on salivation. These prejudices frequently deprive us of the use of this invaluable article: an article which we can use when scarcely any other can be retained in the stomach; which, when combined with opium, has no superior in calming an irritable stomach; which will change the secretions of the liver, and chylopœtic vicera, from a morbid to a healthy kind, more certainly than any other in the materia medica.

Humanity requires that we should inflict no more pain, nor subject our patients to no more inconvenience or suffering, than the necessity of the case absolutely demands. Why should we then subject our patients to the pain and inconvenience of a mercurial sore mouth, when we are able to cure them without it; especially if we can obtain all the remedial agency of mercury without salivation?

If avoiding every kind of acid during the use of mercury, will enable our patients to escape salivation, by reversing the rule in those cases in which we wish a speedy salivation, we may almost always succeed in producing this effect, by giving acid drinks freely, and at the same time washing the mouth with vinegar, or other acids. This I have found true in those *few* cases in which I have had occasion to try it. And I have very little doubt that other practitioners, after a fair trial, will find it generally the case.

I have been led to make these remarks, in consequence of not being able to recollect any notice of the effect of acids, when given along with, or shortly after the use of, calomel.—Should you think them of any use to the profession, you are at liberty to publish them. Very respectfully, your friend,

THOS. TOWNSEND.

*Massilon, Stark Co. Ohio*, *Sep.* 16*th*, 1830.

Art.—VI. *An account of some sporadic Cases of Apthous Sore Mouth, occurring in Pregnant Females, and attended with some unusual Symptoms.* By Dr. John Cook Bennet, of Barnesville, Ohio.

Believing it to be the duty of every physician, to record and report all diseases attended with malignant or uncommon symptoms, I proceed to give you an account of a very troublesome disease, several cases of which have lately fallen under my observation, and were attended with a degree of obstinacy and severity, that I was perfectly unprepared for by any thing I had previously seen or read.

The subjects of the disease were all females in a state of pregnancy. Whether this circumstance had an influence in predisposing to the disease, or whether the association was merely accidental, the number of cases I have met with does not authorize me to determine. The first symptom which attracted the attention of the patient, was an immoderate flow of saliva, as profuse as if the salivary glands were acted upon by mercury. The saliva soon became so offensive to the patient, as to keep the stomach in a state of continual irritation, and in the more severe cases, to produce anorexia, and occasional vomiting. The tongue soon became coated in the centre, and of a fiery red upon its margin. In a few days, small white vesicles appeared on the tip and edges of the tongue. These vesicles soon extended over the whole surface of the mouth, and were so much disposed to ulcerate, that deep and troublesome sores were formed, so very irritable that scarcely any thing could be taken into the mouth without causing extreme pain. The disease soon extended to the alimentary canal, its progress being marked by a burning sensation in the stomach, with tenderness on pressure, flatulence, acidity, and disposition to vomit, in some instances so great as to reject with violence every thing received into the stomach. The administration of cathartics produced watery evacuations, frequently combined with membranous flakes of coagulable lymph, or with streaks of blood, and in the advanced stages of the disease, with pus. The irritation in some cases appeared to extend to the schneiderian membrane, as both the taste and smell were very much impaired.

The febrile symptoms were very slight in the early stages of the disease, except in one case, in which they ran high. When, however, the patient became reduced from the salivation, pain, and loss of sleep, the vascular system partook in the general irritability. The salivation and the apthous condition of the mouth continued about four months, and then gradually subsided. Upon one case, a troublesome dysuria attended.

In the treatment of the disease, mild purgatives, principally castor oil and magnesia, were used at the commencement, and occasionally, as the symptoms required, throughout the disease, and were almost the only remedies that proved of decided utility. The various antacids were used with very little advantage. The great irritability and soreness limited us very much in the use of gargles. The aqueous solution of opium, produced temporary palliation ; the various astringent gargles were tried withot affording relief. The most advantage was derived, in the advanced stage of the disease, from a weak preparation of muriatic acid. The above with the occasional administration of an anodyne, as the severity of the pain and the irritability of the system required, was the outline of the remedial treatment pursued. The diet of the patient at the commencement, was composed principally of mucilaginous drinks, and in the advanced stages, to these were added light articles of farinaceous food.

Five of my patients recovered, and one died. I am not aware that any mode of treatment was materially instrumental in shortening the disease. It appeared obstinately to run its course, in spite of our remedies, continuing about four months in each case, and then gradually subsiding.

